# The role of RNA regulators, quorum sensing and c‐di‐GMP in bacterial biofilm formation

**DOI:** 10.1002/2211-5463.13389

**Published:** 2022-03-13

**Authors:** Manuel Condinho, Beatriz Carvalho, Adriana Cruz, Sandra N. Pinto, Cecília M. Arraiano, Vânia Pobre

**Affiliations:** ^1^ Instituto de Tecnologia Química e Biológica António Xavier Universidade Nova de Lisboa Oeiras Portugal; ^2^ iBB‐Institute for Bioengineering and Biosciences (IBB) Instituto Superior Técnico Lisboa Portugal; ^3^ i4HB‐Institute for Health and Bioeconomy Instituto Superior Técnico Lisboa Portugal

**Keywords:** biofilms, cyclic diguanylate, quorum sensing, ribonucleases, RNA, small non‐coding RNAs

## Abstract

Biofilms provide an ecological advantage against many environmental stressors, such as pH and temperature, making it the most common life‐cycle stage for many bacteria. These protective characteristics make eradication of bacterial biofilms challenging. This is especially true in the health sector where biofilm formation on hospital or patient equipment, such as respirators, or catheters, can quickly become a source of anti‐microbial resistant strains. Biofilms are complex structures encased in a self‐produced polymeric matrix containing numerous components such as polysaccharides, proteins, signalling molecules, extracellular DNA and extracellular RNA. Biofilm formation is tightly controlled by several regulators, including quorum sensing (QS), cyclic diguanylate (c‐di‐GMP) and small non‐coding RNAs (sRNAs). These three regulators in particular are fundamental in all stages of biofilm formation; in addition, their pathways overlap, and the significance of their role is strain‐dependent. Currently, ribonucleases are also of interest for their potential role as biofilm regulators, and their relationships with QS, c‐di‐GMP and sRNAs have been investigated. This review article will focus on these four biofilm regulators (ribonucleases, QS, c‐di‐GMP and sRNAs) and the relationships between them.

## Graphical abstract

Quorum Sensing, cyclic diguanylate, small non‐coding RNAs and ribonucleases all have an impact on biofilm formation. These regulators are interconnected and operate in consortium to promote bacterial life‐cycle changes between planktonic cells and biofilm communities. Understanding these regulators and their connections is essential to develop novel anti‐biofilm therapeutics that surpass the effect of existing antimicrobial compounds.

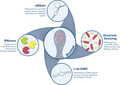

**Abbreviations**
AIs: autoinducers
c‐di‐GMP: cyclic diguanylate
CF: cystic fibrosis
CT: cholera toxin
DGCs: diguanylate cyclases
eDNA: extracellular DNA
eRNA: extracellular RNA
Orn: Oligoribonuclease
PDEs: phosphodiesterases
pGpG: 5‐phosphoguanylyl‐(3′‐5′)‐guanosine
PNAG: poly‐*N*‐acetylglucosamine
QS: quorum sensing
RNases: ribonucleases
sRNAs: small non‐coding RNAs
T3SS: type III secretion system
T6SS: type VI secretion system
TA: toxin‐antitoxin

Bacterial lifestyle is dependent on environmental conditions and the bacterial capacity to adapt to ecosystems. Biofilms are a form of bacterial social behaviour that involves the formation of aggregates of one or more species, which confer extra protection when microbes encounter harsh environments. Biofilms can be attached to a living or non‐living surface [1], and the sessile lifestyle promotes genetic and metabolic diversification of microorganisms [2]. In the biofilm form, bacterial cells are embedded in a self‐produced polymeric matrix consisting of polysaccharides, proteins, signalling molecules, extracellular DNA (eDNA), extracellular RNA (eRNA), and other components [3, 4, 5]. The biofilm matrix is largely dependent on its bacterial species and provides structural stability and protection to the biofilm [3, 6, 7]. The composition of the biofilm matrix also affects the microenvironment, since it determines the biophysical and biochemical properties of the biofilm [3, 5, 6]. Biofilms with mixed‐species are predominant in most environments, but single‐species biofilms are more common in infections and on the surface of medical implants [8].

The formation of biofilms depends on the bacterial species' and/or the nutritional conditions [9, 10], and it is mostly driven by adaptive responses to environmental conditions [11]. This process usually follows a biological cycle that includes attachment, growth, maturation and detachment of the biofilm (Fig. 1) [5, 8]. It begins with the reversible attachment of planktonic cells to a surface suitable for growth [1, 10] following the detection of environmental conditions that trigger a sessile lifestyle [8]. This is followed by (a) irreversible attachment of the cells, (b) growth and (c) the formation of microcolonies surrounded by the biofilm polymeric matrix [3, 10]. These initial attachment phases include cell‐cell and cell‐surface interactions that allow the development of the biofilm [5]. As the bacterial colonies expand, the microorganism occupies the non‐colonised spaces and covers the entire surface [8]. When nutrients are scarce or waste products accumulate, the last step of the cycle starts and bacteria begin to detach from the surface [11]. This can be achieved by (a) downregulating the production of biofilm matrix components, (b) secretion of matrix‐degrading enzymes or (c) disruption of non‐covalent interactions between matrix compounds [11, 12, 13]. Finally, some cells disperse from the sessile structure in a planktonic fashion to colonise other surfaces.

**Fig. 1:**
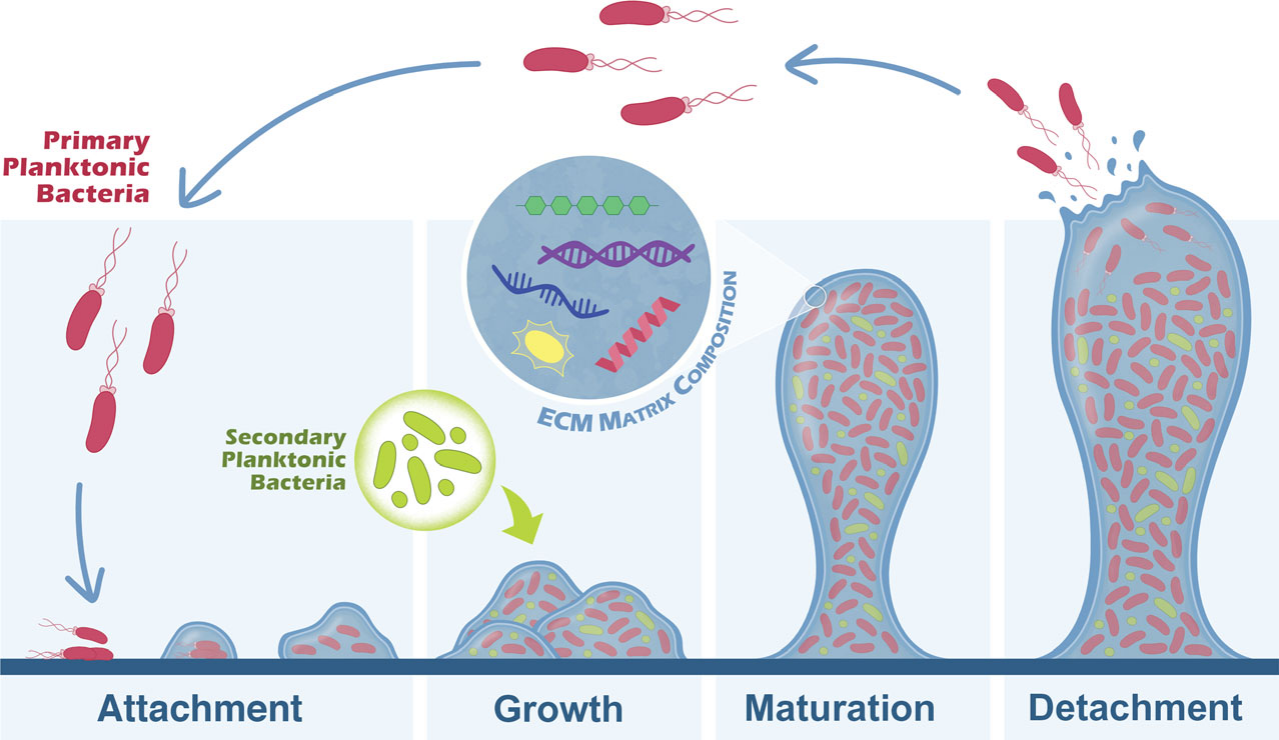
Schematic representation of biofilm formation. Biofilm formation starts with the initial reversible attachment of bacterial cells to a surface. This is followed by the growth of the biofilm within a matrix, maturation of the biofilm, and finally, when the environment conditions cease to be ideal, the reversal of the attachment with the dispersion of the cells that will colonize other surfaces. The biofilm extracellular (EC) matrix is composed of polysaccharides (green), eDNA (purple), eRNA (blue), proteins (red) and signalling molecules (yellow).

Biofilms are extremely difficult to eradicate since they are a community of bacteria engulfed by a protective matrix. This is especially problematic for human health since the biofilms have a higher tolerance to antibiotics. The reduced penetration of antimicrobial agents into biofilms, the occurrence of persister cells, reduced growth and biofilm‐specific protective stress responses all contribute to the observed increased tolerance [6, 14, 15]. Considering that medical devices such as catheters and implants are a major source of infections due to biofilm attachment [16], it is essential to understand the regulatory mechanisms that lead to the formation of biofilms, so that we can develop novel strategies to fight biofilm‐related infections.

This review will focus on the regulation of biofilms by RNA regulatory mechanisms [small non‐coding RNAs (sRNAs) and ribonucleases (RNases)], quorum sensing (QS) and cyclic diguanylate (c‐di‐GMP), all of which are important for all stages of biofilm formation, in particular, to the initial steps. Furthermore, the possible links between these regulators will be explored.

## RNA regulatory mechanisms that affect biofilm formation

The switch from planktonic to biofilm formation is a complex process and is dependent on RNA regulators. There are two classes of RNA regulators that are known to control the formation of biofilms: sRNAs and RNases.

sRNAs are regulatory molecules that control gene expression in cells; several examples of sRNAs that regulate biofilms are already known. On the other hand, RNases are enzymes that process and degrade all types of RNA, but they are less studied as biofilm regulators.

### Small non‐coding RNAs

In their natural habitats or in an infection context, bacteria are constantly exposed to different environmental conditions and stress exposure. To detect, survive and respond to stress, several bacterial mechanisms have evolved to regulate gene expression. sRNAs are regulatory elements that control physiological and metabolic processes and are involved in responses to different stress conditions, such as starvation, hypoxia, antibiotic treatment and high salinity, among others [17, 18, 19]. sRNAs are important regulators of gene expression in bacteria, influencing, either positively or negatively, mRNAs and proteins. When they bind to target transcripts they connect by sequence complementarity, which leads to changes in mRNA translation, stability or both. In fact, base‐pairing between sRNAs and their target mRNAs modifies the accessibility of RNases and/or ribosome binding sites, thereby influencing gene expression [20, 21]. The action of sRNAs often depends on the RNA chaperone Hfq that can facilitate and stabilize the interactions between the sRNAs and their target mRNAs [20, 22]. Hfq has pleiotropic effects, and it is decisive for many sRNA‐mediated regulation pathways, as its deletion affects the stress response, virulence, and biofilms in several bacteria [23, 24]. Some of these non‐coding RNAs are also able to interact with proteins, altering their function/conformation or blocking their binding sites to other nucleic acids [25]. It is increasingly evident that sRNAs play an important role in pathways of biofilm formation, reprogramming gene expression profiles to promote the transition between a planktonic and a surface‐associated lifestyle, and vice versa (Table 1) [26, 27].

**Table 1:** Examples of sRNAs involved in the formation of biofilms and bacterial pathogenicity.

sRNA	Organism	Target	Effect on Target	Phenotype	References
CsrB, CsrC	*Escherichia coli*	CsrA	Repression	Biofilm formation. motility inhibition	[28, 29]
McaS		CsgD	Repression	Curli synthesis decrease. Flagella synthesis promotion	[62]
RprA			Repression	Adhesive curli fimbriae downregulation	[63]
OmrA/OmrB			Repression	Curli synthesis decrease	[64]
GcvB			Repression	Biofilm formation inhibition	[65]
RydC			Repression	Curli synthesis decrease. Biofilm formation inhibition	[66]
RybB			Repression	Biofilm formation inhibition	[67]
DsrA		RpoS	Activation	EPS synthesis promotion. Antibiotic resistance	[176]
GlmY/GlmZ	Enterohemorrhagic *Escherichia coli* (EHEC)	LEE4/LEE5	Repression	Expression of curli adhesin. Biofilm formation	[177, 178]
DicF		PchA	Activation	Host colonization. Virulence amplification	[179]
PapR	Uropathogenic *Escherichia coli* (UPEC)	PapI	Repression	Inhibition of host tissue adhesion	[180]
ErsA	*Pseudomonas aeruginosa*	AlgC	Repression	EPS synthesis downregulation. Biofilm formation inhibition	[52]
RsmY, RsmZ		RsmA	Repression	EPS synthesis	[43]
HmsB	*Yersinia pestis*	HmsHFRS, HmsD, HmsT	Activation	c‐di‐GMP and EPS increase. Biofilm formation	[148]
sRNA0426	*Streptococcus mutans*	GtfB, GtfC, CcpA	Activation	EPS synthesis. Biofilm formation	[59]
Teg41	*Staphylococcus aureus*	αPSM	Activation	αPSM toxin upregulation	[181]
RNAIII		α‐hemolysin	Activation	Exotoxin upregulation	[182]
		Coa, Rot	Repression	Tissue adhesion dowregulation	[183, 184]
RsaA		MgrA	Repression	Cell surface protein expression. Biofilm formation	[71]
LhrC	*Listeria monocytogenes*	LapB, OppA, TcsA	Repression	Host immune response evasion	[185, 186, 187]
InvS	*Salmonella enterica* serovar Typhimurium	PrgH	Activation	Invasion of epithelial cells	[188]
InvS		FimZ	Repression	Invasion of epithelial cells	[188]

**Fig. 2:**
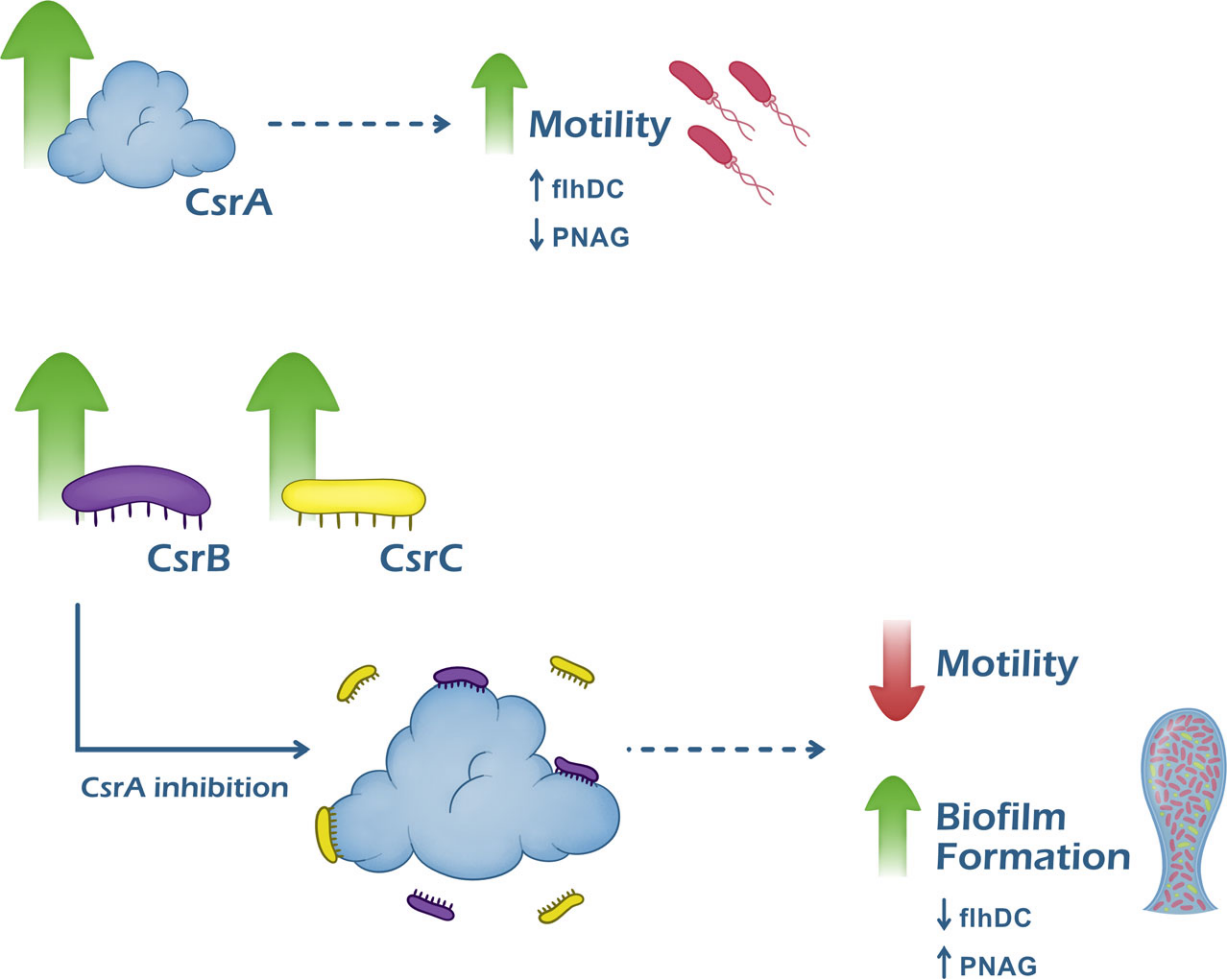
Schematic representation of the Csr regulatory system and its influence on biofilm formation. The protein CsrA stimulates motility by promoting the expression of FlhDC and suppresses the synthesis of the polysaccharide adhesin PNAG. CsrB and CsrC sRNAs sequester and inhibit the CsrA protein, leading to a decrease in bacterial motility and the promotion of biofilm formation.

The *Escherichia coli* sRNAs CsrB and CsrC sequester and inhibit the CsrA protein [28, 29]. CsrA is a key negative regulator of biofilm formation, since it suppresses the synthesis of the polysaccharide adhesin poly‐*N*‐acetylglucosamine (PNAG), and simultaneously stimulates motility by promoting the expression of FlhDC, an important regulator of flagellum biosynthesis (Fig. 2) [30, 31, 32]. As expected, deletion mutants of these two sRNAs in *E. coli* K12 lead to a decrease in biofilm formation since the levels of CsrA are higher [29, 32].

The Csr regulatory system is highly conserved among many pathogenic bacteria, including *Pseudomonas aeruginosa*, *Salmonella* Typhimurium and *Yersinia pseudotuberculosis,* where it controls biofilm formation and virulence mechanisms [33, 34, 35]. Surprisingly, a recent study comparing *E. coli* C and *E. coli* K12 discovered that the levels of CsrB and CsrC are much higher in the K12 strain than in the C strain [36]. This is an apparent contradiction, since *E. coli* C is naturally much more prone to produce biofilms than the *E. coli* K12 strains and, as such, it would be expected that the levels of these sRNAs would be higher in the C strain. In fact, the authors found that in *E. coli* C, there is no compensatory regulation similar to the one that exists between CsrB and CsrC in *E. coli* K12 strains [36]. This difference between two *E. coli* strains is a clear example that although the same systems are conserved among bacteria it should not be assumed that their function is equal. In *P. aeruginosa*, the Csr system is called the Rsm system, and it also regulates the switch between motility and acute infection to sessile lifestyle and chronic infection [33, 37, 38]. Similar to its its *E. coli* homolog (CsrA), RsmA promotes motility, influencing acute infection by positively affecting the expression of the type III secretion system (T3SS) [39]. Furthermore, RsmA seems to repress the production of exopolysaccharides Pel and Psl, which are fundamental components of *P*. *aeruginosa* biofilm [39, 40]. For the establishment of chronic infection, the sRNAs RsmY and RsmZ are fundamental as they sequester RsmA, promoting exopolysaccharide synthesis and inhibiting T3SS production [41, 42, 43]. This, in turn, leads to the production of type VI secretion system (T6SS) and biofilm formation as seen in cystic fibrosis (CF) patients [44, 45]. Other sRNAs of *P. aeruginosa* involved in biofilm formation have also been described [46, 47, 48]. SrbA is an sRNA that is found upregulated in *P. aeruginosa* strain PA14 biofilm cultures, and its deletion results in a 66% reduction in biofilm mass [49]. Similarly, it has also been observed that RgsA sRNA expression is increased in biofilm and that its deletion makes *P. aeruginosa* PAO1 more susceptible to oxidative stress, suggesting it has an important role in the high resistance to this stress frequently observed in bacterial biofilms [46, 50]. ErsA is an sRNA that appears to be involved in the response to envelope stress, which is a pathway that is often related to virulence and biofilm development [37, 51]. This sRNA negatively regulates the expression of the AlgC enzyme, which participates in the biosynthesis of various polysaccharides including alginate, Pel, Psl, LPS, and rhamnolipids, all of which are essential components of the biofilm of *P. aeruginosa* [52]. In this way, ErsA promoted motility, having a relevant role in *P. aeruginosa* pathogenicity during acute infection and in the stimulation of the host inflammatory response [53].

The exopolysaccharide Cepacian, present in biofilms, is a very important component for the efficiency of the infections detected in CF since it protects the bacterial pathogens from antimicrobial treatment and increases their virulence [54, 55]. Several sRNAs have been shown to be relevant for virulence and biofilm formation in *Burkholderia cenopacia*, an opportunistic pathogen also responsible for several persistent lung infections. In *B*. *cenopacia*, Sass and colleagues identified 123 putative sRNAs that are differentially expressed during biofilm formation. The majority of sRNAs were found to be more abundant in biofilms than in *B*. *cenopacia* planktonic cells [56]. The ncS35 sRNA is perhaps the most characterized in this bacterium and is upregulated in biofilm forms that grow in minimal medium. The deletion of ncS35 increased the *B*. *cenopacia* susceptibility to tobramycin, and promoted metabolic activity and the alteration of the biofilm structure, making the bacteria more vulnerable to stress conditions [57].

*Streptococcus mutans* is another microorganism with high relevance regarding biofilm production. This bacterium is the main causative agent of dental caries in humans, and the formation of biofilm is the virulent property that underlies the well‐known dental plaque on tooth surfaces [58]. It was demonstrated that some sRNAs have a positive role in the colonization and biofilm formation of *S. mutans*, thus contributing to its pathogenicity. The *S*. *mutans* sRNA0426 sRNA is overexpressed in biofilms and it is positively correlated with exopolysaccharide production. It was observed that the increase in sRNA0426 may be related with the upregulation of three predicted mRNA targets (GtfB, GtfC, and CcpA) which are involved in the synthesis of exopolysaccharides [59]. Additionally, through deep‐sequencing RNA, it was found that regulation by sRNAs may play a role in the adhesion of *S*. *mutans*, with a total of 736 candidate sRNAs differentially expressed during this process. From this work, two sRNAs (sRNA0187 and sRNA0593) stood out, and their differential expression was confirmed in clinical isolates of *S*. *mutans* [60].

However, there are also sRNAs, which have an opposite action and stimulate motility/repress biofilm formation under specific conditions [26, 27]. In *E. coli*, the transcriptional regulator CsgD is a key player in the complex regulatory circuit that decides whether bacteria form biofilms, since it is necessary for production of curli fimbriae and for the downregulation of several flagellate biosynthesis genes [61]. The expression of *csgD* mRNA is regulated at the translational level by at least seven Hfq‐dependent sRNAs (McaS, RprA, OmrA/OmrB, GcvB, RydC and RybB), which are activated in response to different environmental/stress conditions [62, 63, 64, 65, 66, 67].

*Staphylococcus aureus* is an opportunistic human pathogen capable of leading to bacterial infections in the skin, respiratory tract, and other tissues [68, 69, 70]. RsaA sRNA promotes chronic persistence, biofilm formation and expression of cell surface proteins of *S. aureus*. The main target of RsaA is the *mgrA* mRNA, and RsaA binds to the Shine‐Dalgarno and coding sequence. In this manner, it prevents the formation of the ribosomal initiation complex [71]. In turn, MgrA is a protein that inhibits biofilm formation by suppressing the expression of surface proteins and the release of eDNA [72].

The involvement of sRNAs in the formation of biofilms and pathogenicity has also been described in other microorganisms, such as *Listeria monocytogenes*, and *Helicobacter pylori*, among others (as previously reviewed in [68, 73, 74, 75]).

### Ribonucleases

Ribonucleases are enzymes involved in RNA processing and degradation mechanisms [76]. They are divided into two main classes: exoribonucleases, which cleave RNA one nucleotide at a time, from one extremity and endoribonucleases, which cleave RNA internally. As RNA degrading enzymes they affect the levels of all RNA molecules in the cells, ultimately influencing all cellular processes, including biofilm formation. Thus, they can be considered/explored in the future as novel anti‐biofilm targets [77]. However, only a few very specific examples have been described and their role in controlling biofilms must be further explored.

Oligoribonuclease (Orn) is a 3′ to 5′ exoribonuclease highly conserved in bacteria, but the study of its involvement in biofilms is limited to *P. aeruginosa*, in which it was found that a deletion mutant for Orn cannot degrade 5‐phosphoguanylyl‐(3′–5′)‐guanosine (pGpG) [78, 79]. pGpG is the result of c‐di‐GMP degradation and there is a feedback loop between pGpG and c‐di‐GMP. High levels of pGpG leads to the inhibition of the degradation of c‐di‐GMP, resulting in the accumulation of this second messenger and therefore affecting biofilm formation [80]. Exoribonucleases analogous to Orn (NrnA, NrnB, and NrnC) in *Bacillus anthracis* and *Vibrio cholerae* (a Gram‐negative bacterium that causes cholera) were also shown to affect biofilm formation by hydrolysing pGpG [81].

RNase Y, an endoribonuclease, was also found to affect biofilm formation in *Bacillus subtilis* and *Clostridium perfringens* [82, 83]. In *B. subtilis*, transcriptomic data showed that RNase Y deletion leads to the repression of more than 350 transcripts from biofilm‐related pathways. Moreover, overexpression of RNase Y induced biofilm formation in spotted agar plates [82]. This effect of RNase Y in *B. subtilis* biofilm is probably because this enzyme degrades sinR, which is a repressor of the biofilm matrix genes [84]. In *C*. *perfringens*, RNase Y affects biofilm formation since it stabilizes pilA2 (a pilin component of the type IV pili), which is involved in cell attachment [83]. Deletion of RNase Y in *C*. *perfringens* decreased attachment of cells to surfaces, and consequently affected biofilm formation [83].

In *Mycobacterium tuberculosis*, there is an endoribonuclease, Rv2872, that is also a toxic protein from a toxin‐antitoxin (TA) system. RNA‐Seq data showed that RV2872 affects several transcripts involved in biofilm formation; however, it appears that the effect on biofilms is due to the TA system and not its ribonuclease activity [85].

RNase I is an endoribonuclease of the RNase T2 superfamily and was found to affect *E. coli* biofilm formation since it degrades cytoplasmic RNA to generate 2′,3′‐cNMPs [86]. Using a transposon mutagenesis analysis, another study reported that deletion of an RNase T2 family protein affected the ability of *Acinetobacter baumannii* to attach to surfaces, therefore impairing biofilm formation [87].

RNase J2 has both an exoribonucleolytic and endoribonucleolytic activity and was found to affect the expression of the ebpABC operon that encodes pili proteins that play a major role in biofilm formation in *Enterococcus faecalis* [88].

The exoribonucleases RNase II, RNase R and PNPase were also found to affect biofilm formation in *E. coli*, but while deletion of RNase II and RNase R increase the ability of *E. coli* to form biofilms, the deletion of PNPase completely abolished the capacity of this bacterium to form biofilms [89], as demonstrated by quantification using the crystal violet method. The exact mechanisms by which these exoribonucleases impact on *E. coli* biofilms are still not known, but transcriptomic data showed that several motility, flagellum and biofilm transcripts are significantly altered in the absence of these enzymes [89, 90]. In *S*. Typhimurium, the PNPase deletion mutant was also found to form less biofilm than the wild‐type, but surprisingly, the RNase II mutant formed even less biofilm than the PNPase mutant [91]. Furthermore, the endoribonucleases RNase E and RNase III also seem to affect biofilm formation in *S*. Typhimurium [91], and although the mechanism underlying this phenotype is not known, it has been reported that RNase E affects QS in *Sinorhizobium meliloti* [92], opening a pathway to be further explored. These differences observed might suggest that the effects of RNases in biofilm formation are dependent on the studied microorganism.

Several studies showed that RNases affect biofilm production, but most studies simply demonstrated a phenotypic result, and the underlying mechanism by which this occurs is still not understood.

## Quorum sensing

Quorum‐sensing is a mechanism of cell‐to‐cell communication used by bacteria and involves the production and release of signalling molecules termed autoinducers (AIs). The concentration of signalling molecules increases with bacterial population density. When a minimal threshold concentration of these AIs is reached, bacteria respond by regulating population behaviour, such as with virulence and/or biofilm formation [93].

Biofilm formation induction by QS signals depends largely on the bacterial species present in each biofilm. QS systems differ in terms of the chemical classes to which the QS molecules belong: the acyl homoserine lactones (AHLs), furanosyl borate diesters (AI2), cis‐unsaturated fatty acids (DSF family signals) and peptides [13, 94]. A QS system is comprised of a synthase that produces the autoinducer and the receptor for that specific inducer.

Bacteria usually have more than one QS system; for instance *P*. *aeruginosa* has three QS systems (las, rhl and pqs) that are interconnected [95] and *S*. *aureus* has the Agr system and the *luxS* gene that produces AI‐2 [13]. However, high concentrations of AIs do not always induce the formation of biofilms. In fact, there are two distinct types of response: the positive, where at high cell density AIs accumulate and bacteria respond by forming biofilms, and the negative response that occurs when AIs accumulation represses biofilm formation [96].

*P. aeruginosa* is one of the most well‐studied organisms in terms of the effects of QS in biofilm formation. This bacterium responds positively to the AI concentration in the environment, meaning that high levels of signalling molecules will promote biofilm formation. These signalling molecules were found to affect the production of several components of the biofilm matrix, such as the polysaccharide Pel and Psl [97, 98], rhamnolipids [99] and eDNA [100].

*Vibrio cholerae* also has three QS molecules: CAI‐1 ((S)‐3‐hyroxytridecan‐4‐one) synthesized by CqsA, AI‐2 synthesized by LuxS, and DPO, synthesized by Tdh [101]. In contrast with observations made for *P. aeruginosa*, high levels of these AIs will repress the formation of *V*. *cholerae* biofilm. The QS systems of *V*. *cholerae* are greatly interconnected with sRNAs and this will be further described in Interconnections between the key biofilm regulators of this review.

In *S. aureus*, there are two main QS regulatory systems: (a) the accessory gene regulator (Agr) system and, (b) the LuxS system. The *S*. *aureus* Agr system controls the biofilm detachment process by promoting the expression of several small amphipathic peptides [102, 103]. This system is controlled by an RNA regulator, the RNAIII sRNA [104]. Both the Agr QS components and RNAIII are in the same chromosome region and under the control of the P2 promoter [105]. The Agr regulon is comprised of several hundred genes and most of these are indirectly regulated via RNAIII [104, 106]. Agr has different, important roles in biofilms as it can control virulence determinants, including regulation of *S*. *aureus* toxins [107], and can have an impact on acute infection and toxicity [108]. The LuxS system is less studied, but there is also evidence that it impacts on the expression of biofilm genes essential for exopolysaccharide biosynthesis [109]. Furthermore, *in vitro* and *in vivo* studies showed that *luxS* can control *S. aureus* biofilm growth through the *icaR* locus [110].

Due to the importance of QS in biofilms, there are several therapeutic approaches proposed for targeting it [108, 111, 112].

## C‐di‐GMP

The secondary messenger bis(3′,5′)‐cyclic diguanylic acid (c‐di‐GMP) is ubiquitous in nature. It has a role in several bacterial signaling pathways, and its pleotropic action impacts a diverse set of cellular players. In particular, there is a well‐established link between c‐di‐GMP signaling, bacterial virulence and biofilm formation. Over the years, insightful studies have shown that c‐di‐GMP is involved in the spread of bacteria in the host, evasion/subversion of immune defense mechanisms and in the colonization of tissues [113]. Changes in c‐di‐GMP levels were shown to allow/facilitate the transition between bacterial lifestyles, where an increase in c‐di‐GMP level correlates with a switch from an active, fast‐spreading and motile lifestyle to a slow‐growing biofilm lifestyle [114]. During this process, the presence of c‐di‐GMP promotes the biosynthesis of adhesins and exopolysaccharides, and inhibits processes related to motility such as the functioning of the flagellar motor [115].

Intracellular levels of c‐di‐GMP are maintained by two types of enzymes: the diguanylate cyclases (DGCs), which synthesize c‐di‐GMP from two GTP molecules, and the phosphodiesterases (PDEs) that degrade c‐di‐GMP in pGpG. While the GGDEF conserved domain is essential for DGC enzymatic function, PDE activity is mainly attributed either to their EAL or HD‐GYP domains [114, 116]. Given their determinant control over c‐di‐GMP levels, DGCs and PDEs play an important role in biofilm formation and virulence of bacteria [117, 118, 119, 120, 121, 122]. For instance, deletion of the gene for BifA, a PDE expressed in both *P*. *aeruginosa* and *Pseudomonas putida*, results in severe defects in motility and a hyperbiofilm phenotype given the general increase in c‐di‐GMP levels [123, 124]. In some *Pseudomonas* species, WspR is a DGC that enhances the synthesis of c‐di‐GMP and suppresses the T3SS, leading to increased exopolysaccharide production that is readily observed by the formation of wrinkly colonies [118, 125, 126]. The suppression of T3SS also leads to the upregulation of T6SS, which is associated with biofilm formation and chronic infections of *P. aeruginosa* [45]. In *P*. *aeruginosa*, c‐di‐GMP also acts on Alg44, FleQ and PelD proteins, regulating the synthesis of alginate, Pel and Pls polysaccharides. These exopolysaccharides are important for the formation of the extracellular matrix of bacteria, acting as a shield against antibiotics [40, 122, 127, 128]. Alginate is particularly relevant in persistence of *P. aeruginosa* in several diseases including CF, contributing to pathogenic roles such as inhibition of phagocytosis, suppression of neutrophil chemotaxis and scavenging of oxidative radicals [129, 130].

In *E*. *coli* K‐12, YddV and YdeH are two DGCs that are necessary for the expression of PNAG, an exopolysaccharide that is present in a wide variety of bacteria biofilms [131, 132]. In particular, the expression of YdeH is upregulated in response to antibiotics, leading to a strong biofilm induction [132]. Furthermore, the YhjH PDE plays an important role in adherent‐invasive *E. coli* in Crohn disease, by promoting flagellar function and type 1 pili synthesis, and thereby enabling the invasion of the host's intestinal epithelial cells [133]. In this bacterium it was also described that the transcription factor BolA controls the expression of several DGCs and PDEs, thereby affecting the levels of c‐di‐GMP and consequently biofilm formation [134]. It was shown that the balance between these two factors is important for the accurate regulation of the transition between the planktonic and sessile lifestyles. This balance is achieved by negative‐feedback regulation of BolA and c‐di‐GMP. However, even in the presence of elevated c‐di‐GMP levels, biofilm formation is reduced in the absence of BolA [134].

In humans, *V*. *cholerae* causes cholera by colonizing the small intestine and secreting cholera toxin (CT) [135]. During infection by this bacteria, c‐di‐GMP signaling plays an important role in virulence, since low intracellular levels of this molecule promote the production of CT. It was observed that, during this process, the PDE VieA is required to keep the concentration of c‐di‐GMP low, and thus enhance colonization in an animal model of infection [136]. Moreover, VpsT, a transcription factor and a master regulator of biofilm formation in *V*. *cholerae* is affected by high levels of c‐di‐GMP [137, 138]. Biofilm formation is of particular importance for this bacterium since it allows the colonization of humid environments, as well as resistance to low pH and chemical stress [139].

*Klebsiella pneumoniae* is an opportunistic Gram‐negative bacterium, whose pathogenicity increases with the ability to form biofilms, which in turn promotes microbial colonization of host tissues [140]. The transcription factor MrkH regulates the production of type 3 fimbriae, a organelle that allows adherence to human endothelial and urinary bladder cells [141]. MrkH‐mediated expression of type 3 fimbriae is enhanced by the presence of c‐di‐GMP [142]. Moreover, c‐di‐GMP promotes the expression of MrkH, creating positive feedback that stimulates the formation of biofilms in *K*. *pneumoniae* [143].

Additionally, some DGCs and PDEs which are enzymatically inactive can recognize c‐di‐GMP and function only as effectors of this secondary messenger. For instance, *Pseudomonas fluorescens* expresses LapD, an inner‐membrane protein required for biofilm formation and the maintenance of the adhesin LapA. Secretion of LapA is dependent on the binding of c‐di‐GMP to the degenerate EAL domain of LapD, determining the surface commitment of *P*. *fluorescens* [144].

Various effectors, from enzymes to transcription factors, which are directly or indirectly involved in biofilm formation, are sensitive to changes in c‐di‐GMP levels [113]. The PilZ family of proteins is the best described group of c‐di‐GMP effectors, since the PilZ domain was the first to be identified as binding specifically to c‐di‐GMP [145, 146]. In *E. coli* and *Salmonella enterica*, YcgR, a PilZ domain protein, impairs motility in response to high levels of c‐di‐GMP. Under these conditions, YcgR interacts with the flagellar switch‐complex proteins, reducing the motor function, thereby facilitating the transition from motile to a sessile/biofilm lifestyle [147, 148]. Another PilZ domain protein of *E. coli* is BcsA, which upon c‐di‐GMP binding, stimulates the synthesis of cellulose, a common component of this bacteria biofilm [149]. However, it is important to bear in mind that not all c‐di‐GMP effectors possess a PilZ domain [150].

The broad range of mechanisms by which c‐di‐GMP affects biofilm formation is due to its capacity to bind to several proteins allosterically and change their structure and/or function. Alternatively, c‐di‐GMP can also interact with nucleic acids, such as mRNA or small regulatory RNA molecules, to regulate gene expression at a post‐transcriptional level [116].

## Interconnections between the key biofilm regulators

There are relevant data concerning the link between three of the main regulators of biofilms (QS, c‐di‐GMP and sRNA), but the link between exoribonucleases and the other regulators has not yet been explored in depth.

*Vibrio cholerae* provides a good example of interconnection between QS, sRNAs and c‐di‐GMP. As mentioned above, in this microorganism there are three QS systems, and high levels of AI repress biofilm formation. This occurs because at low levels of CAI‐1 and AI‐2, the QS receptors (CqsS, CqsR, LuxPQ and VpsS) act as kinases and promote the phosphorylation of the LuxO response regulator. In this phosphorylated state, LuxO activates four small regulatory RNAs (Qrr sRNAs), which promote the expression of genes necessary for biofilm formation [13, 101]. When the cell density increases with a consequent increase in AIs, the QS receptors conversely act as phosphatases; LuxO is subsequently dephosphorylated, which thereby represses biofilm formation and causes *V*. *cholerae* to disperse from the existing biofilms [13, 101]. At high cell density, the DPO autoinducer also binds to the VqmA receptor, which in turn activates the expression of the sRNA VqmR that represses biofilm formation [101]. Moreover, the repression of biofilm formation by these high levels of AIs occurs through an extensive network of genes, including 14 genes encoding proteins that synthesize and degrade c‐di‐GMP [151].

There are many examples of connections between two regulators of biofilm formation, but the interconnection of sRNAs and QS is the most well studied. For example, deletion of the *Agrobacterium tumefaciens* sRNA AbcR1 promotes the import of Gamma‐aminobutyric acid (GABA) that in turn promotes the degradation of the QS signal N‐(3‐oxo‐octanoyl) homoserine lactone [152]. In *P*. *aeruginosa* the sRNA PhrS binds to the short upstream open reading frame of the pqsR gene, stimulating its translation and increasing the synthesis of quinolone signal molecules [153]. Furthermore, *E. coli* microarray data suggests that the CyaR sRNA negatively regulates *luxS*, and this was experimentally validated by northern blot [154]. QS can also regulate sRNAs. For instance, the sRNA MicA is close to the genomic location of the *luxS* gene in *S*. Typhimurium, and deletion of *luxS* CDS leads to a significant decrease in the levels of this sRNA [155]. There are several other examples of the interconnection of sRNAs and QS systems, which have been extensively reviewed in [156].

There are also several studies linking sRNAs and c‐di‐GMP. For example, the sRNAs CsrB and CsrC regulate the protein CsrA that binds to mRNAs of the DGCs, *ycdT* and *ydeH*, repressing their translation and decreasing c‐di‐GMP levels [131]. Similarly, in *P*. *putida* RsmA, which is regulated by two sRNAs, RsmY and RsmZ, was shown to affect c‐di‐GMP levels through the response regulator CfcR [157]. More complex regulation occurs during the biofilm development of *Yersinia pestis*, a bacterial agent that causes bubonic plague, using fleas as a vector [158]. In this microorganism the existence of a stable extracellular biofilm matrix enhances bacterial aggregation before the bacteria spread through the host skin and lymphatic systems. The *hmsHFRS*, *hmsD*, *hmsT* and *hmsP* genes encode the major factors involved in biofilm formation of *Y*. *pestis* [158]. The first is an operon responsible for biosynthesis and translocation of biofilm matrix exopolysaccharide. HmsD and HmsT are DGCs responsible for the synthesis of c‐di‐GMP, and HmsP is a PDE that degrades c‐di‐GMP [158, 159]. Interestingly, the HmsB sRNA regulates all these factors, stimulating the expression of *hmsHFRS*, *hmsD* and *hmsT*, and inhibiting the expression of *hmsP*. This leads to increased production of c‐di‐GMP and exopolysaccharides, making HmsB one of the main activators of biofilm formation in *Y*. *pestis* [148]. Additionally, c‐di‐GMP also affects the expression of sRNAs; for example, in *Dickeya dadantii*, a plant pathogen, a mutation of the DGC *gcp*A resulted in increased RNA levels of the RsmB sRNA [160]. Another example is found in *V*. *cholerae* where the putative sRNA P1‐Vc2 was found to be increased in direct proportion with c‐di‐GMP levels [161]. The interconnection between sRNA and c‐di‐GMP may be much more widespread than is currently believed, since Hfq was found to affect the levels of c‐di‐GMP in *Y*. *pestis* and *D*. *dadantii* [162, 163]. Since most sRNAs depend on Hfq to bind to their targets there might be a link between Hfq, sRNAs and c‐di‐GMP that is yet to be explored. A recent review on Hfq relationship with c‐di‐GMP showed that there are several avenues of research that need to be pursued to uncover these complex regulatory systems.

Another relationship that needs to be considered is the connection of RNases with c‐di‐GMP and QS systems. In fact, the exoribonuclease PNPase is known to be activated by c‐di‐GMP [164] and it is possible that the regulation of biofilm by this exoribonuclease (described in Ribonucleases) is correlated with c‐di‐GMP. Moreover, PNPase affects sRNA metabolism [165, 166] and it is also possible that the effects observed in the absence of this enzyme are due to its role on sRNAs that control biofilm formation. Similar to PNPase, RNase E is also known to degrade several sRNAs, and most notably it degrades both CsrB and CsrC, indirectly affecting c‐di‐GMP levels [167]. Additionally, the Orn regulates c‐di‐GMP levels in several organisms since it is involved in pGpG metabolism [78, 79, 81]. There is at present not much information regarding the connection of RNases with QS systems. It is known that RNase E and RNase J affect the S‐adenosylmethionine (SAM) methyl donor which is involved in the AHLs QS system in *S*. *meliloti* [168]. The exact mechanism by which they affect QS in this organism is still not fully understood, but RNase E also degrades sinI, a gene encoding the acyl‐homoserine lactone synthase [92]. Another example linking QS with RNases involves the TA modules. Several toxins from these TA also have endoribonucleolytic activity, namely MazF, ChpBK and MqsR toxins. In *E. coli*, activity of both MazF and ChpBK is stimulated by the QS pentapeptides NNWNN (which are called EDFs‐extracellular death factors) [169], and the EDFs also enhance the activity of MazF in *M. tuberculosis* [170]. MqsR is induced by the AI‐2 QS signal [171], but it is not known how this affects its endoribonucleolytic activity.

In addition to the above, much is known regarding the connection of the QS with the c‐di‐GMP regulatory machinery. Kozlova et al. showed that the link of QS systems with c‐di‐GMP is relevant for the regulation of virulence in *Aeromonas hydrophila* [172]. The authors showed that c‐di‐GMP overproduction modulated the transcriptional levels of genes involved in biofilm formation and motility phenotype in a QS‐dependent way that involved the AI‐1 and AI‐2 systems. In a different study, c‐di‐GMP was observed to induce expression of *aphA* in *V*. *cholerae* [173]. *aphA* is an activator of virulence gene expression and an important QS regulator [174]. These two biofilm regulators have been extensively studied, and a more comprehensive analysis of their connection can be reviewed in [175].

**Fig. 3:**
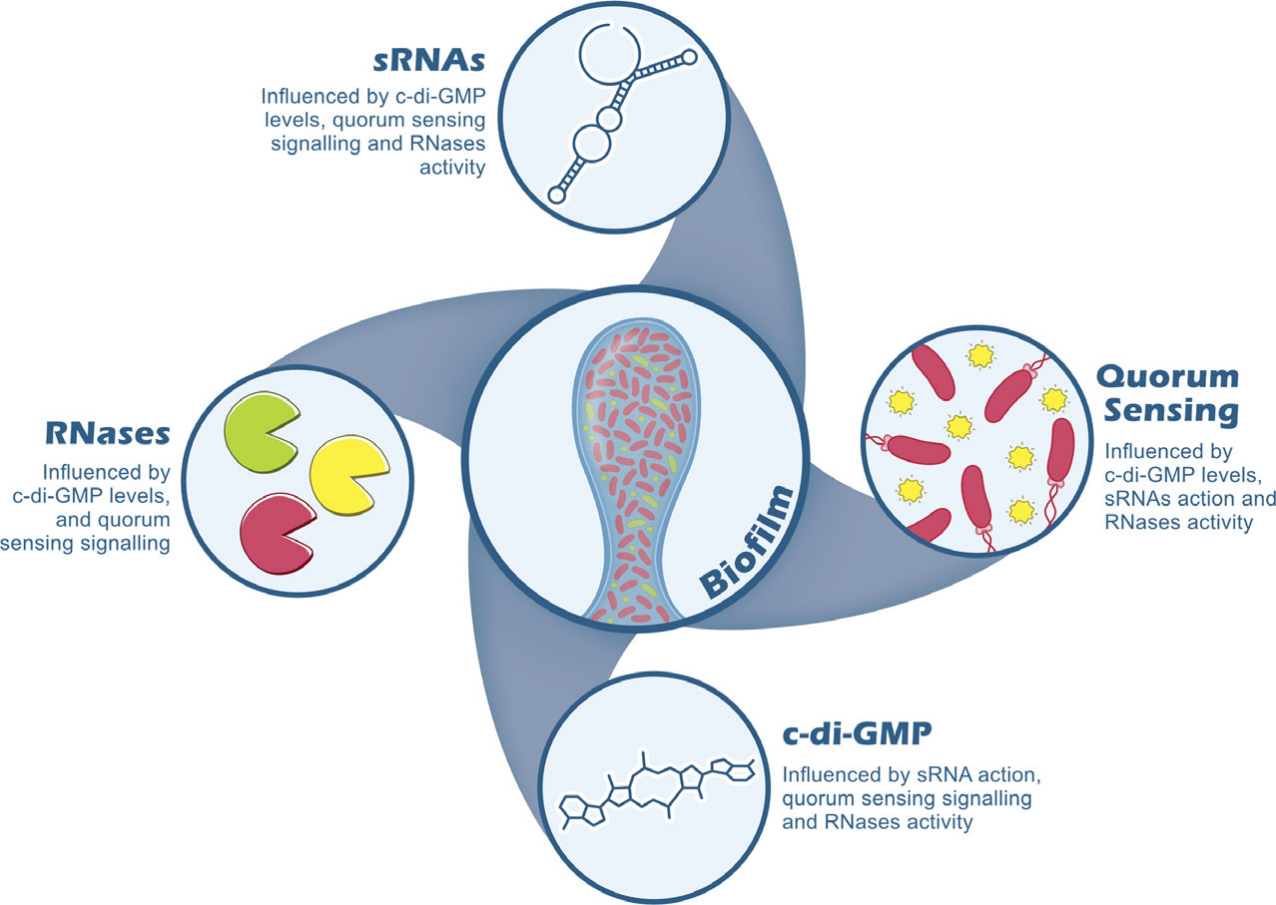
Schematic representation of the important regulators of biofilm formation. QS, c‐di‐GMP, sRNAs and RNases all have an impact on biofilm formation. The connections among all these regulators leads to the promotion or repression of bacterial biofilms and is also important for the maintenance of mature biofilms.

Recently, we have published an extensive review of the latest antibacterial and antibiofilm strategies [77]. Most novel strategies being developed target QS and c‐di‐GMP, due to limited understanding of how RNA regulators may be harnessed for biofilm control. It is however clear that the four regulators highlighted in this review have interconnected roles in biofilm formation (Fig. 3) and these complex interactions should be taken into consideration when developing new strategies for biofilm disruption.

## Concluding remarks

Biofilms are complex structures that give bacteria a great advantage to survive under stress conditions. In fact, biofilms are the predominant lifestyle for most bacteria, and this has serious consequences for human health since the protective characteristics of biofilms make it hard to efficiently eradicate biofilm‐related infections. This is particularly troubling in hospital settings where the growth of biofilms in medical devices frequently leads to the emergence of multidrug resistance strains. It is therefore essential to develop novel strategies to fight bacteria when they exist in these communities, highlighting the importance of studying the regulatory mechanism behind biofilm formation. In this review, we gave a small overview of the current knowledge on the three main regulators of biofilms: sRNAs, QS and c‐di‐GMP. Additionally, we also examined a lesser‐known RNA regulator of biofilms, the RNases, to bring attention to this avenue of research exploring their impact on biofilm formation. There is sufficient evidence suggesting these regulators are interconnected and operate in consortium to promote bacterial life‐cycle changes, as illustrated in Fig. 3. Further research in this area will hopefully further elucidate the how these regulators relate to each other. This will promote the discovery of innovative anti‐biofilm therapeutics that surpass the effect of existing antimicrobial compounds.

## Conflict of interest

The authors declare no conflict of interest.

## Author contributions

Conceptualization—SNP, VP; writing—MC, AC, BC, CMA, SNP, VP; supervision—SNP, VP. All authors have read and agreed to the published version of the manuscript.
